# Fractal Structure of Brain Electrical Activity of Patients With Mental Disorders

**DOI:** 10.3389/fphys.2022.905318

**Published:** 2022-07-13

**Authors:** Dick O. E, Murav’eva S. V, Lebedev V. S, Shelepin Yu. E

**Affiliations:** ^1^ Laboratory of Physiology of Reception, Pavlov Institute of Physiology of Russian Academy of Science, St. Petersburg, Russia; ^2^ Laboratory of Vision Physiology, Pavlov Institute of Physiology of Russian Academy of Science, St. Petersburg, Russia

**Keywords:** multifractality, singularity spectrum, brain activity, schizophrenia, depression

## Abstract

This work was aimed at a comparative analysis of the degree of multifractality of electroencephalographic time series obtained from a group of healthy subjects and from patients with mental disorders. We analyzed long-term records of patients with paranoid schizophrenia and patients with depression. To evaluate the properties of multifractal scaling of various electroencephalographic time series, the method of maximum modulus of the wavelet transform and multifractal analysis of fluctuations without a trend were used. The stability of the width and position of the singularity spectrum for each of the test groups was revealed, and a relationship was established between the correlation and anticorrelation dynamics of successive values of the electroencephalographic time series and the type of mental disorders. It was shown that the main differences between the multifractal properties of brain activity in normal and pathological conditions lie in the different width of the multifractality spectrum and its location associated with the correlated or anticorrelated dynamics of the values of successive time series. It was found that the schizophrenia group is characterized by a greater degree of multifractality compared to the depression group. Thus, the degree of multifractality can be included in a set of tests for differential diagnosis and research of mental disorders.

## 1 Introduction

Despite the huge number of works devoted to the study of the nonlinear dynamics of the bioelectrical activity of the brain in various pathologies (Slezin et al., 2007; Suckling et al., 2008; Mukli et al., 2018; Racz et al., 2020; Lee et al., 2021; Racz et al., 2021; Alamian et al., 2022), the identification of neurophysiological markers of these pathologies remains an extremely urgent task. This is especially true for diseases associated with cognitive impairment such as Alzheimer’s disease, schizophrenia, epilepsy, and the goal of such work is not only to obtain new theoretical data and understanding of pathophysiology, but also to use these data to improve clinical diagnosis, assess the severity or progression of the disease.

At the same time, changes in the bioelectrical activity of the brain can be associated with both oscillatory and fractal brain functions. Changes in oscillatory activity imply changes in the frequency range of the electroencephalographic (EEG) time series in the pathological brain compared to the healthy brain. For example, a number of studies report an increase in the amplitude of the EEG delta range in patients with schizophrenia compared with the control group (Knott et al., 2001; Harris et al., 2006). Other studies (Begić et al., 2000; Harris et al., 2001; John et al., 2009) have shown that different phenotypes of schizophrenia can be characterized by both a decrease and an increase in the amplitude of the delta range, depending on the positive or negative forms of schizophrenia. In addition, treatment with neuroleptics (Harris et al., 2006; John et al., 2009) or an increase in the duration of the disease (Harris et al., 2001; Tislerova et al., 2008; John et al., 2009; Ranlund et al., 2014) can lead to a decrease in delta activity.

However, oscillatory processes with characteristic frequencies (delta, theta, alpha, and beta oscillations) in the EEG also exhibit fractal (scale-free) behavior. In this case, the EEG oscillatory power spectrum containing these characteristic frequencies is superimposed with a fractal spectrum in which the power is inversely proportional to frequency, and the relationship is established through a power function with the scaling factor β (Eke et al., 2002).

Using irregular-resampling auto-spectral analysis (IRASA) (Wen and Liu, 2016), it is possible to separate the power spectrum into its two components, i.e., extract the spectrum of oscillatory activity without the confounding effects of broadband activity, and the spectrum of the fractal component of the signal with subsequent estimation its spectral scaling exponent β, which will not be evaluated by the presence of oscillatory peaks.

In (Racz et al., 2021), this method was used to answer the question of whether the differences in power spectra found between healthy subjects and patients with schizophrenia are associated with changes in the fractal or oscillatory components of the EEG. The authors of (Racz et al., 2021) showed that the amplitude of the delta range in the initial power spectrum is reduced for patients with schizophrenia compared with the control group, mainly in the central regions of the brain; however, this difference could be attributed almost exclusively to a shift of power towards higher frequencies in the fractal component.

The differences found in the initial spectra were present only in the fractal component of the spectrum, but not in the oscillatory one. Thus, the authors of (Racz et al., 2021) came to the conclusion that the differences in EEG patterns between healthy and diseased brains are not necessarily associated exclusively with changes in the rhythmic component of neuronal activity, but necessarily with the broadband fractal component of this activity. This is another confirmation of the importance of fractal analysis for neurophysiological rhythms.

It is important to note that a number of studies have shown that many physiological rhythms associated with movement, the work of the heart and brain have multifractal properties (Ivanov et al., 1999; Watters and Martin, 2004; Acharya et al., 2005; Nurujjaman et al., 2009; Sassi et al., 2009; Scafetta et al., 2007; Scafetta et al., 2009; Dick, 2017). This is explained by a paradoxical combination of short-term decorrelation caused by noise and long-range correlation caused by the fractal structure of these rhythms (Ivanov et al., 1999; Scafetta et al., 2007). It means that their patterns on small scales are not identical to the whole time series but the self-similarity remains after averaging by statistically independent samples of the time series (Pavlov and Anishenko, 2007).

Multifractality of the healthy brain is revealed in electroencephalographic (EEG) time series during complex imaginary and real visual-motor task performance (Popivanov et al., 2006; Wink et al., 2008), during awake and various sleep stages (Qianli et al., 2006). Multifractality of the EEG time series is found also during epileptic discharges (Song and Lee, 2005; Dick and Mochovikova, 2011; Dick and Svyatogor, 2012) and in neural disorders connected with anxious phobia combined with headache, tachycardia or disturbance of the breathing rhythm (Dick and Svyatogor, 2012).

Note that the method of estimating multifractal scaling properties of EEG time series combined with searching the rate of the change of the modulus of the wavelet coefficients of the EEG, so called wavelet transform modulus maxima method (WTTM) (Muzy et al., 1993), allows one to establish structural adjustments leading to a change in multifractal properties, i.e. reveal the mechanisms of dynamic changes in the structure of EEG patterns in the event of a particular pathological condition (Dick and Svyatogor, 2012; Dick and Nozdrachev, 2019).

Another method for assessing the multifractality of a signal is multifractal detrended fluctuation analysis (MFDFA) (Kantelhardt et al., 2002). This method was generalized from detrended fluctuation analysis applied for monofractals (Peng et al., 1994). Currently, multifractal analyses are promising prognostic and diagnostic tools in biomedical signal processing (Eke et al., 2000; Eke et al., 2002; Ihlen, 2012).

The multifractal spectrum of endogenous brain dynamics and response times is more sensitive to the influence of age and cognitive performance compared to a single power law exponent alone (Dick and Mochovikova, 2011; Dick and Svyatogor, 2012). In (Suckling et al., 2008; Ihlen and Vereijken, 2010) it was shown that in the absence of epileptic discharges, the EEG dynamics of a patient with focal epilepsy is practically indistinguishable from the EEG dynamics of a healthy brain. But already in the period preceding the epileptiform activity, the EEG dynamics changes and rearrangements occur, leading to the emergence of a correlation of successive EEG values, which is the reason for the increase in the EEG amplitude during an epileptic discharge.

Multifractal analysis also makes it possible to evaluate the effectiveness of the treatment of patients with neural disorders associated with psychogenic pain syndromes. Thus, it was shown in (Dick and Svyatogor, 2012) that variations in multifractal properties explain the changes that occur during psychorelaxation, reflecting the persistence or removal of psychogenic pain in patients with anxious phobic disorders.

The fact is that the width of the multifractal spectrum serves as a measure determining the degree of multifractality of the analyzed time series, since the smaller the width of the spectrum, the closer the tendency to monofractality of the time series (Muzy et al., 1993; Popivanov et al., 2006). The position of the multifractal spectrum is related to the correlation or anticorrelation dynamics of successive values of the time series. The presence or absence of correlations and anticorrelations of successive values of the analyzed time series is determined by the values of Hölder exponents obtained by the WTMM or MFDFA methods (Muzy et al., 1993; Kantelhardt et al., 2002). So if the values of these exponents are in the range from 0 to 0.5, then the dynamics of consecutive values of the time series is anticorrelated, but if the values of these exponents are in the range of 0.5 and higher, then the dynamics of consecutive values is correlated (Pavlov and Anishenko, 2007; Eke et al., 2002). In the first case, there is an alternation of large and small values of the analyzed series (a large value is more likely to be followed by a small one and vice versa). In the second case, a large value is often followed by a large one, and a small value is often followed by a small one, hence the time series is more “smooth”. Therefore, differences in the degree of multifractality and correlation or anticorrelation dynamics can be included in a set of tests for the differential diagnosis of mental disorders.

The purpose of this work is the comparative analysis of the multifractality in EEG patterns of normal and pathological brain activities like schizophrenia and depression and the identification of relationships between the width of the multifractal spectrum, as well as the correlation or anticorrelation dynamics of consecutive EEG values and the type of mental disorders.

## 2 Materials and Methods

### 2.1 Experimental Procedure

The study involved 10 patients with a paranoid form of schizophrenia (F20 according to the ICD 10) with a disease duration from one to 10 years, including 5 men and 5 women aged 24–35 years) and 10 patients with depression (F32; F33 according to the ICD 10), including 5 men and 5 women aged 21–34 years. The control group consisted of 10 healthy subjects (5 men and 5 women aged 18–30 years). All subjects had visual acuity of at least 0.9, and refraction was normal.

Among the symptoms observed in the majority of patients with schizophrenia who participated in the study, it should be noted the predominance of positive symptoms of schizophrenia (auditory hallucinations (voices) and delusions of persecution). Also, these patients were characterized by such symptoms as tension, alertness, anxiety and ambivalence (a dual, contradictory attitude of the subject toward the object, characterized by the simultaneous direction of opposite impulses to the same object, occurring suddenly and regardless of the circumstances).

In the group of the overwhelming majority of depressive patients who participated in the study, signs of vital depression were detected with varying degrees of melancholy manifestation with unreasonable pessimism, despondency and depression. This group was characterized by a circadian rhythm of affect in the form of a distinct deterioration in the morning hours with an improvement in the evening. Many patients complained of a violation of the ability to think logically and to establish consistent connections between events.

Among the common symptoms of patients in the two groups, one can note anxiety and disturbances in the structuring of thinking, as well as a decrease in the ability to concentrate attention.

Both groups of patients (with schizophrenia and depression) were on antipsychotic therapy and took the antipsychotic aripiprazole (Abilify). It is known that this substance has the least effect on EEG power spectra, unlike other known antipsychotic drugs (clozapine, olanzapine and chlorpromazine) (Takashi Ozaki et al., 2021). In connection with the search for differences in the fractal properties of the EEG time series of different groups of patients, it can be considered reasonable to analyze the data when taking the same antipsychotic drug.

To record EEG time series, an encephalograph (Mitsar EEG -202, Russia) with a sampling frequency of 250 Hz and the WinEEG software were used. Recordings were performed using an electroencephalographic cap (ElectroCap, International Inc, United States) with 19 electrodes located on the surface of the head in accordance with the 10–20 International System in the leads Fp1; Fp2; F7; F3; Fz; F4; F8; T3; C3; Cz; C4, T4; T5; P3; Pz; P4; T6; O1; O2. Reference electrodes were placed on the earlobes, and a ground electrode was placed in the frontal region. We analyzed recordings from all sites.

The EEG recordings were obtained under resting condition with eyes closed. Then the data were digitally filtered using 0.5–50 Hz band pass filter. After repeated recordings the segments of equal duration (120 seconds) were tested.

The segments with rough artifacts were eliminated after visual inspection, artifacts due to blinking were eliminated using a procedure for independent component analysis using EEGLAB software (http://www.sccn.ucsd.edu/eeglab/). Artifact-free EEG segments used for analysis consisted of 23,300 samples.

### 2.2 Estimation of EEG Segment Multifractality

To estimate multifractal scaling properties of EEG time series the wavelet transform modulus maxima (WTMM) method (Muzy et al., 1993) and the multifractal detrended fluctuation analysis (MFDFA) (Kantelhardt et al., 2002) were applied.

The continuous wavelet transform of a time series describing the examined signal *x*(*t*) was determined as:

$$W\left(a,t_{0}\right)=\frac{1}{a}\int_{-\infty }^{+\infty }x\left(t\right)\psi^{*}\left(\frac{t-t_{0}}{a}\right)dt,$$

where *a* is the scale parameter, *t*_0_ is the space parameter, *ψ*((*t*-*t*_0_)/*a*) is the wavelet function obtained from the basic wavelet *ψ*(*t*) by scaling (stretching or compressing) and shifting along the time, symbol * means the complex conjugate. So, the wavelet transform of the signal consists in decomposing it into elementary space-scale contributions associated to wavelets which are constructed from one function by means of scaling and shifting.

The complex Morlet wavelet

$$\psi \left(t\right)=\pi^{-1/4}e^{i\omega_{0}t}e^{-t^{2}/2}$$

was used as the basic wavelet, where the value *ω*_0_ = 2π gives the simple relation *f* = 1/*a* between the scale *a* and the frequency *f*:

Information about possible multifractality of the analyzed time series and its localization *t*_0_ reflects in the asymptotic behavior of coefficients |*W* (*a, t*_0_)| at small *a* values and large *f* values, respectively (Bacry et al., 1993). The faster the wavelet coefficients decrease at *f*→∞, the more regular the signal is around the point *t*_0_. The small decrease of the wavelet coefficients at *a*→0 in a neighborhood of the point *t*_0_ testifies about non-regularity or singularity of the signal at the point. Thus, the rate of the change of the modulus of the wavelet coefficients enables to determine the presence or absence of singularities of the signal.

The degree of singularity of the signal *x*(*t*) at the point *t*_0_ is described by the Hölder exponent, *h* (*t*_0_), the largest exponent such that the analyzed signal in a neighborhood of the point *t*_0_ can be represented as the sum of the regular component (a polynomial *P*_*n*_(*t*) of order *n* < *h* (*t*_0_)) and the non-regular component:

$$x\left(t\right)=P_{n}\left(t\right)+c{\left|t-t_{0}\right|}^{h\left(t_{0}\right)},$$

where *с* is a positive constant (Bacry et al., 1993).

The value *h* (*t*_0_) is the measure of singularity of the signal at the point *t*_0_ since the smaller *h* (*t*_0_) value, the more non-regular (more singular) the signal.

In view of the fact that under the condition

$$\int_{-\infty }^{+\infty }t^{m}\psi \left(t\right)dt=0$$

wavelets are orthogonal to polynomials up the degree *m* and for *m* ≥ *n* the expression

$$\int_{-\infty }^{+\infty }P_{n}\left(t\right)\psi \left(t\right)dt=0$$

is true, then the simple power dependence

$$W\left(a,t_{0}\right)∼a^{h\left(t_{0}\right)}$$

is observed at *a*→0 (Arneodo et al., 1995).

Hence, the Hölder exponent can be calculated by the rate of the decrease of the wavelet coefficients by decreasing the scale *a:*

$$h\left(t_{0}\right)∼\frac{\log_{10}W\left(a,t_{0}\right)}{\log_{10}a}.$$

However, by increasing the scale *a*, the influence of neighboring nonregularities can lead to inaccuracy and in practice the Hölder exponents are found on the basis of statistical description of local singularities by partition functions (Muzy et al., 1993; Arneodo et al., 1995) constructed with the WTMM method.

These partition functions are calculated by the sum of *q* powers of the modulus maxima of the wavelet coefficients along the each line at the scales smaller the given value *a*:

Z(q,a)=∑l∈L(a)(sup|W(a∗,tl(a∗))|a∗≤a)q,

where *t*_*l*_ (a*) determines the position of the maximum corresponding to the line *l* at this scale.

By the fact that at *a*→0 the partition function shows the power (Arneodo et al., 1995):

$$Z\left(q,a\right)∼a^{\tau \left(q\right)},$$

the scaling exponent *τ*(*q*) can be extracted as the slope of a log-log plot of the partition function versus the scale *a*:

$$\tau \left(q\right)∼\frac{\log_{10}Z\left(q,a\right)}{\log_{10}a}.$$

Choosing different values of the power *q* one can obtain a linear dependence *τ*(*q*) with a constant value of the Hölder exponent

$$h\left(q\right)=\frac{d\tau \left(q\right)}{dq}=const$$

for monofractal signals or nonlinear dependence

τ(q)=qh(q)−D(h)

with large number of the Hölder exponents

$$h\left(q\right)=\frac{d\tau \left(q\right)}{dq}\neq const$$

describing local scaling of the wavelet coefficients for multifractal signals.

The distribution of the local Hölder exponents (singularity spectrum) is calculated from the Legendre transform (Bacry et al., 1993):

D(h)=qh(q)−τ(q).

The algorithm for estimating signal multifractality using the MFDFA method consists of the following sequence of procedures.

First, for the original series of values ${\left\{x\left(t_{i}\right)\right\}}_{i=1}^{N}$ an integrated sequence is calculated, consisting of the accumulated deviations from the mean $\overset{⌢}{x}$ :

$$y\left(i\right)=\sum_{k=1}^{i}\left(x_{k}-\hat{x}\right),i=1\text{,}...\text{,}N\text{.}$$

This sequence is divided into a number *m* = *N/n* of non-overlapping intervals of length *n*, the partition is repeated, starting from the opposite end, resulting in 2*m* intervals.

For each of the intervals, the resulting sequence is approximated by a straight line using the least squares method, as a result of which the local trend *v*_*s*_(*i*) is determined within the selected interval.

Next, the deviations of the calculated sequences relative to the local trend are determined for each interval *s* = 1,…, *m* и *s* = *m*+1,…, 2*m*:

$$\begin{array}{l} F^{2}\left(n,s\right)=\frac{1}{n}\sum_{i=1}^{n}{\left[y\left(\left(s-1\right)n+i\right)-v_{s}\left(i\right)\right]}^{2},\\ F^{2}\left(n,s\right)=\frac{1}{n}\sum_{i=1}^{n}{\left[y\left(N-\left(s-m\right)n+i\right)-v_{s}\left(i\right)\right]}^{2}, \end{array}$$

and the fluctuation function *F*_*q*_(*n*) *q* order is calculated:

$$F_{q}(n)={\left\{\frac{1}{2m}\sum_{s=1}^{2m}[F^{2}(n,s){]}^{q/2}\right\}}^{1/q}.$$

The calculations are repeated for other values of the interval length n from 5 to 100.

Due to the fact that with an increase in the length of the interval *n*, the value of *F*_*q*_(*n*), as a rule, increases according to a power law:

$$F_{q}\left(n\right)\text{∼}n^{h\left(q\right)},$$

the Hölder exponent *h*(*q*) can be calculated as the slope of the straight line that defines the dependence of log *F*_*q*_ *(n)* от log *n*.

Using the WWTM and MFDFA algorithms one obtain the width of the singularity spectrum

$$\Delta h=h_{max}-h_{min}\text{;}$$

where $h_{\max}=h$ |_*q=q* min_ and $h_{\min}=h$ |_*q=q* max_ are the maximal and minimal values of the Holder exponent corresponding to minimal and maximal fluctuation of the signal, respectively.

The width of the singularity spectrum, Δ*h*, is a measure determining the degree of multifractality of the signal since the small Δ*h* value indicates that the time series tends to be monofractal and the large Δ*h* value testifies the enhancement of multifractality.

We note that when using the WWTM algorithm, the appearance of a distorted singularity spectrum is possible due to the distortion of the shape of the *h*(*q*) curve, as a rule, in the vicinity of values of the moment *q* close to zero.

The work (Mukli et al., 2015) proposes the fan-like convergent geometry of scaling functions yielding a limit value (termed focus) for all moments at the largest scale. Building on this behavior of scaling, the authors of (Mukli et al., 2015) introduced the novel concept of focus-based multifractal formalism. It relies on enforcing this universal behavior when the moment-wise scaling exponents are assessed for the scaling functions. Due to the fact that in the analysis of our data, in 99% of cases, no distortion of the shape of the *h*(*q*) curve was observed, this method was not used.

To examine the differences between the mean values of the width of the singularity spectra obtained for different sites of one subject, the non-parametric Kruskal–Wallis test was used. To compare the mean widths of the singularity spectra obtained for different subjects and to estimate statistical difference for both groups of patients and for the control group, one-way ANOVA followed by multiple comparison and pairwise hypothesis testing using Tukey’s test (Hochberg and Tamhane, 1987) was applied. If the statistics obtained by the Fisher *F*-criterion exceeded the critical value *F*_*crit*_ = *F*_2,28_ = 3.3, then the null hypothesis about the equality of the means was rejected. Values 2 and 28 were chosen based on the fact that the number of groups is *k* = 3, the number of averaged values in each group is 10, the total number of observations is *N* = 10*3 = 30, therefore *k*-1 = 2, N - *k* = 28. Statistically significant differences between groups were determined based on *p* < 0.017 values due to the fact that n = *k* (*k*-1)/2 = 3 and 1–0.951/*n* = 0.017.

## 3 Results

**FIGURE 1:**
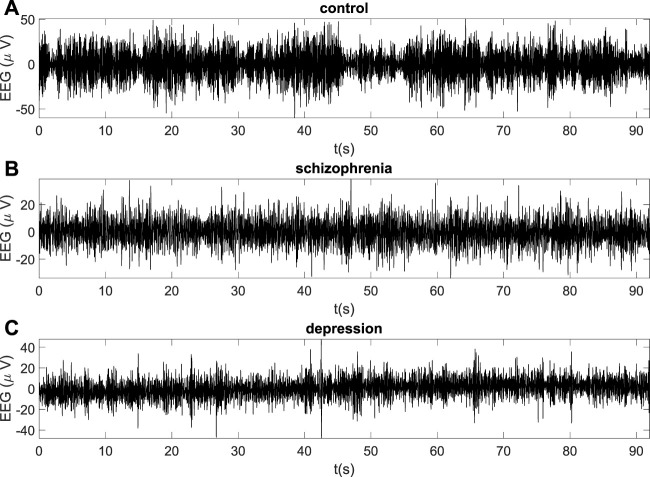
The examples of EEG recordings for the healthy subject **(A)**, the patient with schizophrenia **(B)** and the patient with depression **(C)** (O2 site).

The typical EEG recordings for the healthy subject and the patient with schizophrenia and the patient with depression are represented in Figure 1 (A-C) for frontal O2 site.

**FIGURE 2:**
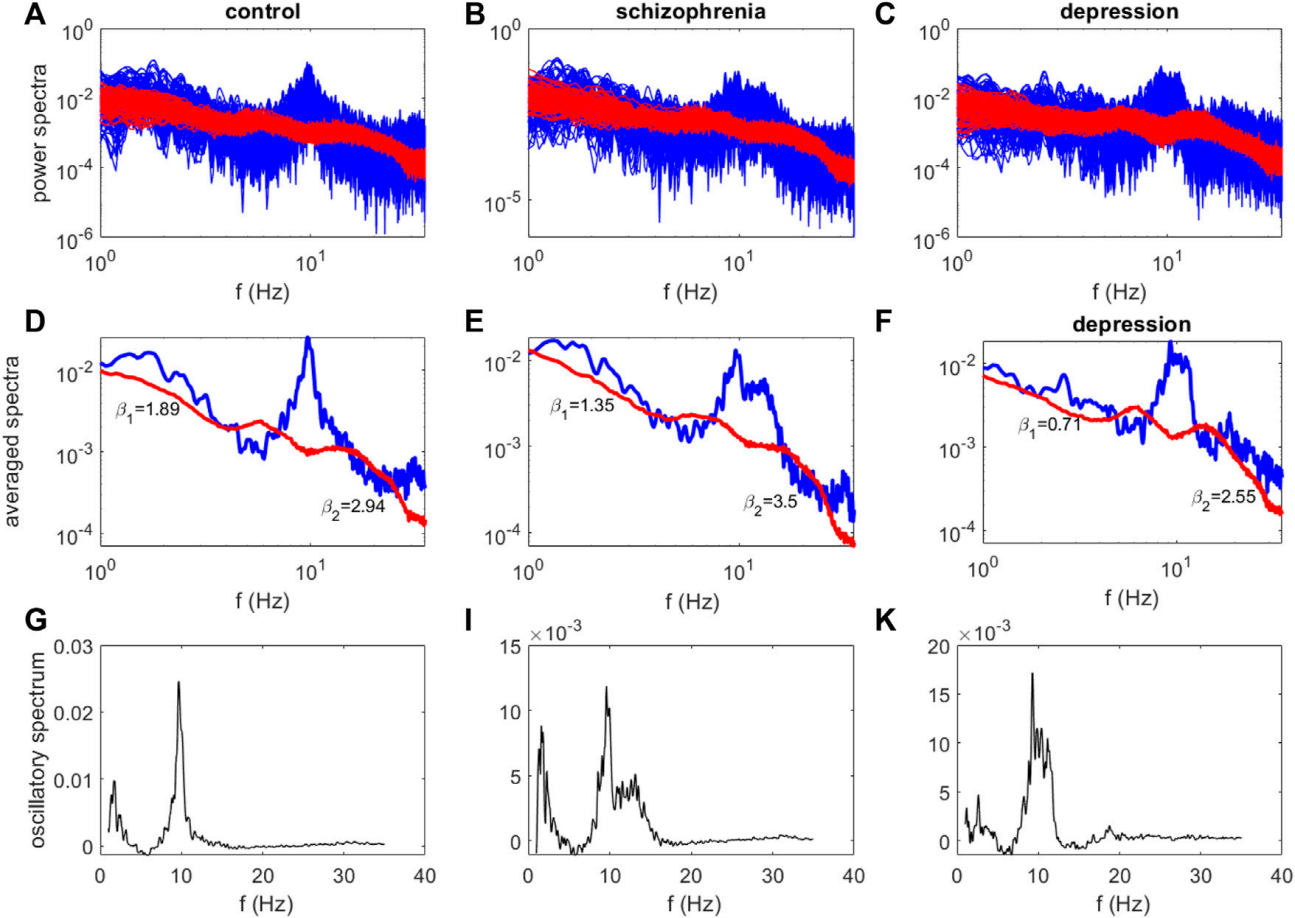
The examples of power spectra for the healthy subject **(A,D,G)**, the patient with schizophrenia **(B,E,I)** and the patient with depression **(C,F,K)** (O2 site). The original (mixed) power spectra are marked in blue, the fractal components - in red **(A–C)**. The averaged mixed and fractal power spectra **(D–F)**, the oscillatory spectra **(G–K)**.

**FIGURE 3:**
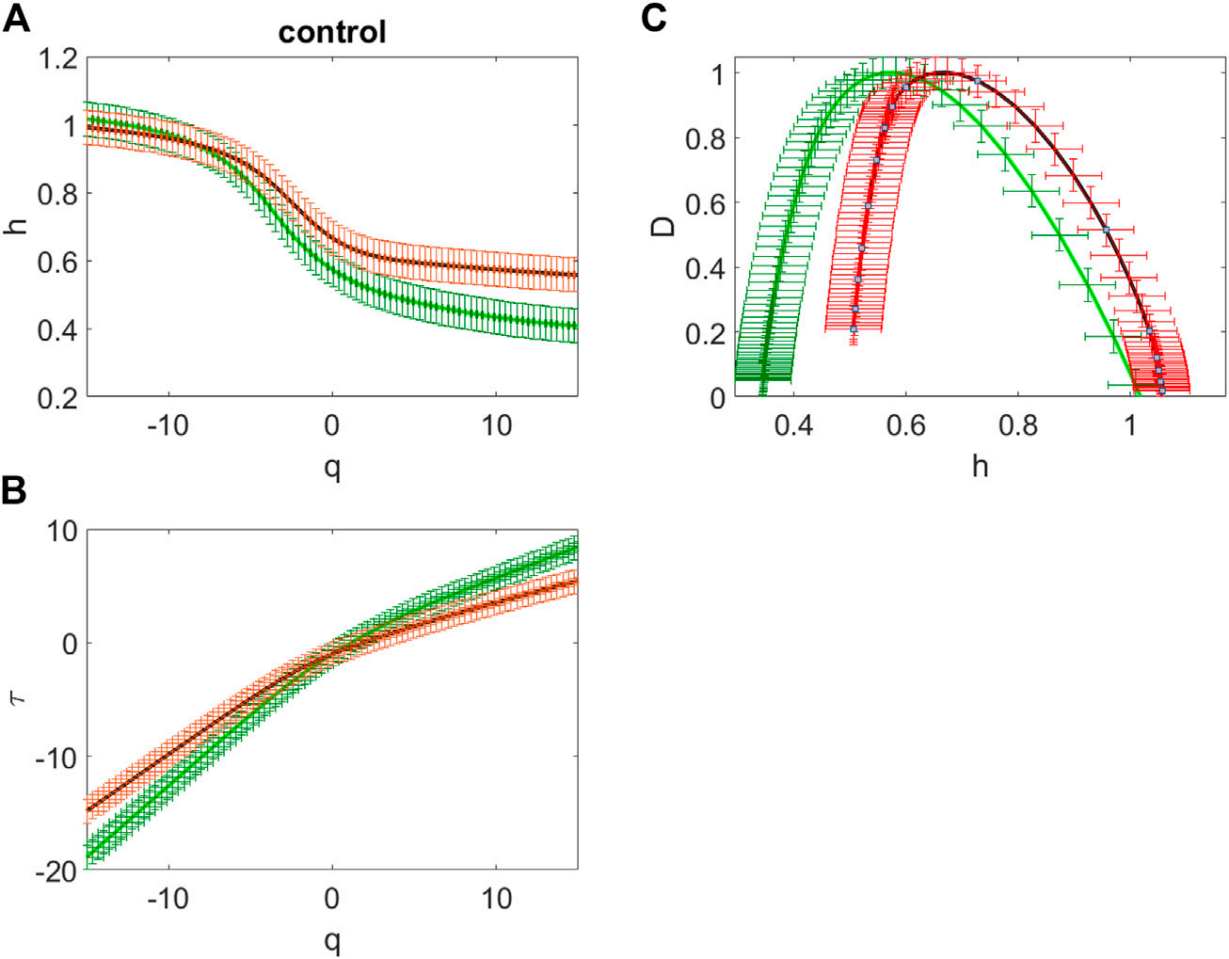
The averaged dependences *h*(*q*) of the Hölder exponent on the power *q* value **(A)**, the averaged scaling exponents τ(q) **(B)** and the averaged singularity spectra **(C)** for the sites over the frontal regions (F3, Fz, F4) (green curves) and over the central (C3, C4), occipital (O1 and O2), parietal (P3, P4 and Pz) and temporal (T5 and T6) regions (red curves) for the subject from the control group.

Figure 2 illustrates examples of power spectra obtained for these subjects using the IRASA method. The original (mixed) power spectra are marked in blue and the separated fractal components are marked in red (Figure 2 A−C). The averaged mixed and fractal power spectra are represented in Figures 2D–F. Despite the presence of similar frequency peaks in the delta and alpha ranges in the gained oscillatory spectra (Figure 2 G—K), the fractal components of the calculated spectra differ (Figure 3 D—F), and the values of their scaling exponents β calculated from these spectra also differ.

By analogy with (Racz et al., 2021), we estimated two the spectral slopes calculated in the two frequency ranges separately, yielding estimates of β_l_ and β_2_ characterizing the slope of the fractal power spectrum in the 1–13 Hz and 13–30 Hz regimes, respectively. The largest value of spectral scaling exponents β_2_ = 3.5 corresponds to the fractal spectrum of a patient with schizophrenia, and the smallest value β_2_ = 2.55 corresponds to the fractal spectrum of a patient with depression. The smallest value of β_1_ = 0.71 was obtained for the fractal spectrum of a patient with depression.

In connection with the well-known relationship between the spectral scaling exponent β and the Hurst exponent β = 2*H* + 1, Hurst exponent values are *H*_1_ = 0.45 and *H*_2_ = 0.97 for the healthy subject and *H*_1_ = 0.17 and *H*_2_ = 1.27 for the patient with schizophrenia and *H*_1_ = -0.14 and *H*_2_ = 0.77 for the patient with depression. This indicates that the correlated dynamics of successive values of the analyzed EEG patterns is most likely characteristic of the healthy subject; for patients with schizophrenia and depression, the dynamics of successive values of patterns is apparently not only correlated but also anticorrelated. A detailed idea of correlations and anticorrelations is provided by the results of multifractal analysis.

Figure 3 gives example of dependences *h*(*q*) of the Hölder exponent on the power *q* value (Figure 3A) and the scaling exponents τ(q) (Figure 3B) and the singularity spectra (Figure 3C) for the subject from the control group obtained using the WWTM method. Green curves correspond to the sites over the frontal regions (Fp1, Fz, F3, F4) and red curves correspond to the sites over the central (C3, C4), occipital (O1 and O2), parietal (sites P3, P4 and Pz) and temporal (sites T5 and T6) regions.

The shape of the represented curves indicates that for all the analyzed sites the given EEG time series actually have multifractal properties. Really, the curves τ(q) are nonlinear and the Hölder exponents *h(q)* are not constant and they depend on the moment *q*. This leads to the fact that the singularity spectra *D*(*h*), depicted in Figure 3 C, display a single humped shape that characterizes intermittent fluctuations corresponding to the Hölder exponent values spanning the interval of width Δ*h* exceeding 0.5. In other words, these spectra are the sets of the multifractal dimensions of the EEG time series.

For the given EEG time series the singularity spectrum is in the range of the Hölder exponents 0.34 < h < 1.05 for the sites over the frontal regions and it is in the range 0.51 < *h* < 1.05 for the other sites (Figure 3C). Therefore, for the healthy subject, the oscillations in this example are characterized by long-term correlations in most areas of the brain. The frontal network is characterized by an expansion of the singularity spectrum and a shift towards anticorrelated dynamics.

**FIGURE 4:**
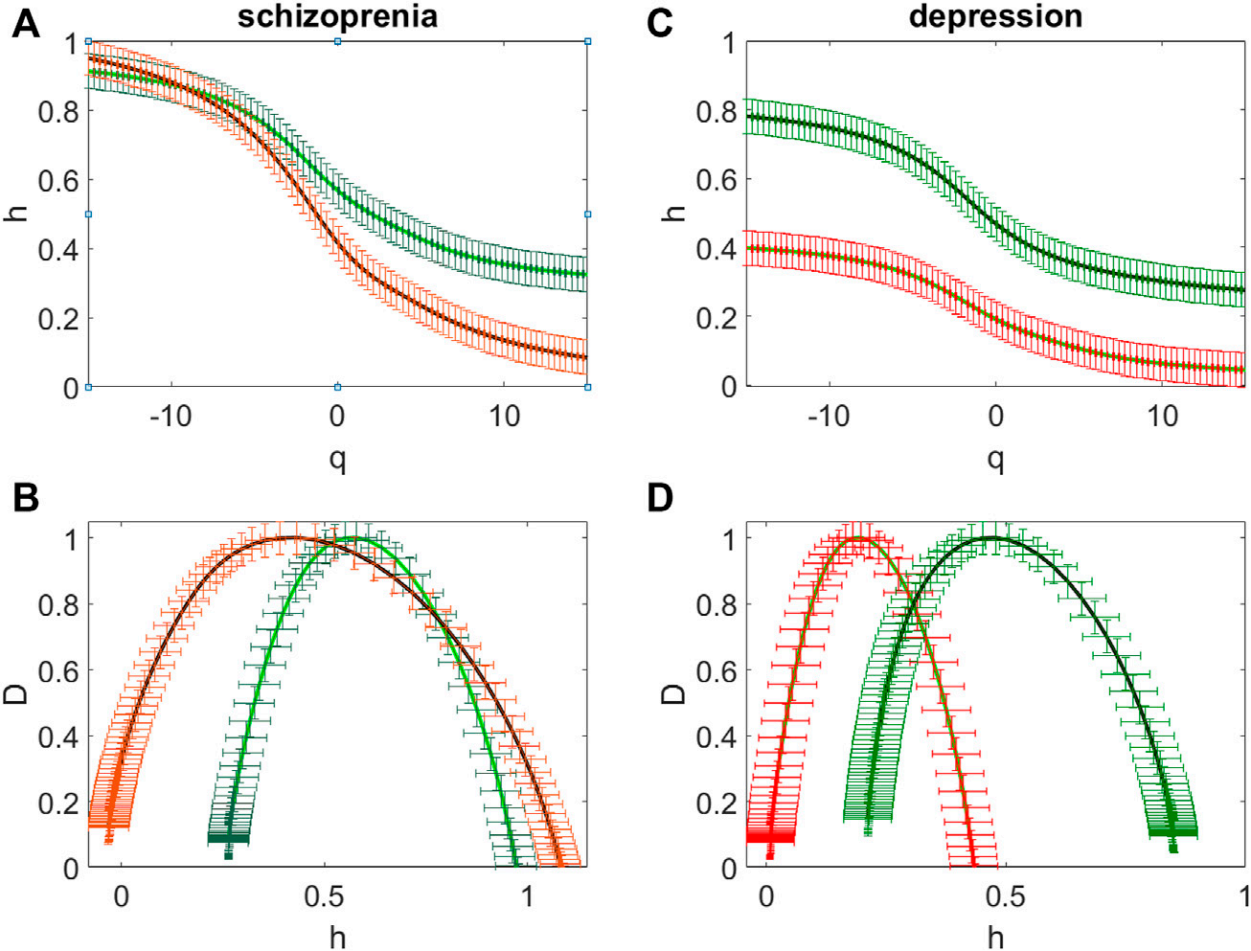
The averaged dependences *h*(*q*) of the Hölder exponent on the power *q* value **(A,C)** and the averaged singularity spectra **(B,D)** for the patient with schizophrenia **(A,B)** and for the patient with depression **(C,D)**. The sites over the frontal and central regions (F3, Fz, F4, C3, C4) (green curves) and the sites over the occipital (O1 and O2), parietal (P3, P4, Pz) and temporal (T5 and T6) regions (red curves).

Figure 4 illustrates multifractal properties of the EEG time series for the patient with schizophrenia (Figure 4A, B) and for the patient with depression (Figure 4 C, D). This is confirmed by the dependence of the Hölder exponents *h(q)* on the moment *q* (Figure 4 A, C). Green color indicates the curves obtained for sites over the frontal regions (F3, Fz, F4), the curves obtained over the central (C3, C4), occipital (O1 and O2), parietal (P3, P4 and Pz) and temporal (T5 and T6) regions are marked in red.

For the patient with schizophrenia the singularity spectrum is in the range of the Hölder exponents 0.26 < *h* < 0.97 for the sites over the frontal (F3, Fz, F4) and central (C3, C4) regions (green curves, Figure 4B) and it is in the range -0.03 < *h* < 1.13 for the other sites (red curves, Figure 4 B).

For the patient with depression for the sites over the frontal and central regions the width of the singularity spectrum and its location are similar to those obtained for the patient with schizophrenia (green curves, Figure 4B), the spectrum is in the range of the Hölder exponents 0.28 < *h* < 0.83 (green curves, Figure 4D). For the other sites the singularity spectrum is in the range 0.02 < *h* < 0.53 (red curves, Figure 4D).

Thus, differences in these spectra are not characteristic of the frontal network (sites F3, Fz, F4) and the somatomotor network (sites C3, C4 and Cz), but they are observed in sites associated with the dorsal attention network (sites P3, P4 and Pz) and with the visual network (sites T5, T6, O1. O2).

Both strong fluctuations (at *q* > 0) and weak fluctuations (at *q* < 0) contribute to this shift to the correlated dynamics of the time series over the frontal and somatomotor networks (green curves Figure 4 A, C), while weak fluctuations dominate for the singularity spectra *D*(*h*) in the sites over the dorsal attention and visual networks because at *q* > 0 values of *h* are close to zero (red curves Figure 4 A, C).

The location of the singularity spectrum in the range of Hölder exponents 0. < *h* < 1.2 for the sites over all regions for the patient with schizophrenia (Figure 4B) corresponds to both anticorrelated (for *h* < 0.5) and correlated (for *h* > 0.5) dynamics of consecutive values of the EEG time series (Eke et al., 2002; Pavlov and Anishenko, 2007). The correlation of successive signal values means that a larger signal value is more likely to be followed by a larger one, and vice versa. Thus, for the long–range correlations the oscillatory process is persistent, i.e. maintaining the tendency, and with the low level of random factors (Arneodo et al., 1995).

Figure 4D shows that the degree of long-term correlations decreases and the spectrum of the singularity turns out to be in the range of Hölder exponents 0. <*h* < 0.5 (red curves Figure 4D) for the patient with depression at the transition from the frontal and somatomotor networks to the dorsal attention and the visual networks. It means that long-term correlations of successive values of the EEG time series almost disappear and the singularity spectra shift to the region of anticorrelated values.

Thus, presented in Figure 3 and Figure 4 data show differences in the spectra of singularities of the EEG time series for subjects from the various analyzed groups.

We note that the power spectra of the control group contained mainly the alpha range in all areas of the brain except for the frontal zone, in which delta range fluctuations were also present. Alpha and delta rhythms were typical for all areas of the brain for the group with schizophrenia, while for the somatomotor and frontal networks the amplitude of the delta rhythm was higher than the alpha rhythm and fluctuations were observed in the beta range. For the depressed group, alpha and delta rhythms were also present in the all sites, while for the visual network the alpha rhythm dominated.

**FIGURE 5:**
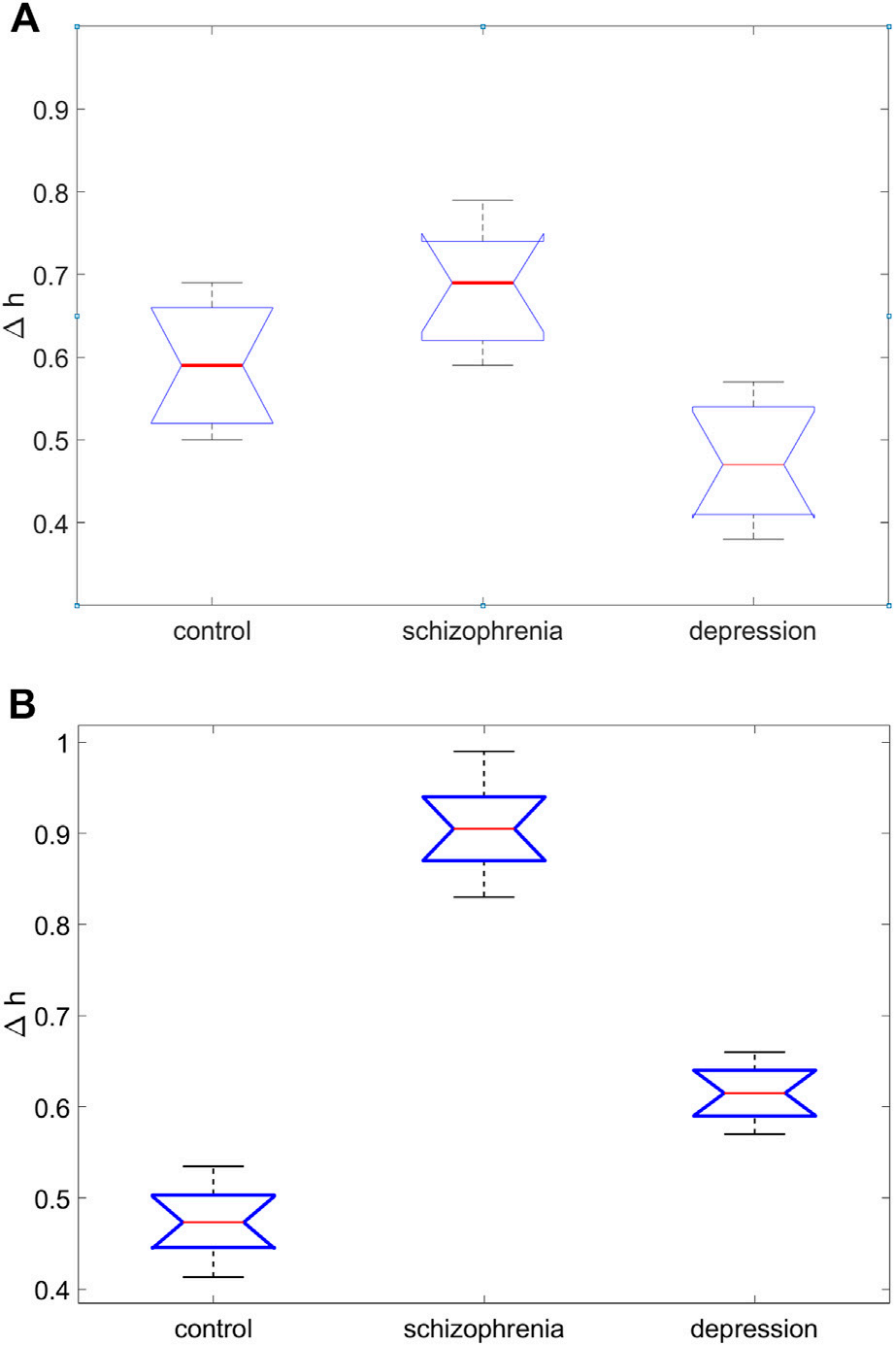
Results of one-way analysis of variance for comparing the average values of the width of the singularity spectrum, ∆*h*, in three groups of subjects for the site C4 **(A)** and the site O2 **(B)**.

Figure 5 illustrates the similarity of the results of multifractal analysis obtained by the WTMM and MFDFA methods. Despite the differences in the width of the multifractality spectrum for each subject (the healthy subject, Figure 5A−Figure 5C and the patient with schizophrenia, Figure 5D—Figure 5F), the region of the spectrum is preserved.

Table 1 illustrates the variability of the averaged (over subjects) maximal and minimal values of the Holder exponent (*h*_min_ and *h*_max_) and the width of the singularity spectrum, Δ*h*, gained by the WTMM and MFDFA methods for O1 site for different groups, and the values of the Fisher test (*F*) and the significance level of the test (*p*), calculated on the basis of one-way analysis of variance. The data obtained by the different methods for the same groups differ slightly.

**TABLE 1:** Comparison of the averaged (over subjects) maximal and minimal values of the Holder exponent (*h*_min_ and *h*_max_) and the width of the singularity spectrum, Δ*h*, for different groups, the values of the Fisher test (*F*) and the significance level of the test (*p*), calculated on the basis of one-way analysis of variance (O1 site).

Parameters	*control group*	*group with schizophrenia*	*group with depression*	*F*	*p*
the MFDFA method					
Δ*h*	0.47 ± 0.05	0.91 ± 0.07	0.58 ± 0.06	276	7*10^−19^
*h*_min_	0.56 ± 0.02	0.04 ± 0.01	0.11 ± 0.03	332	8*10^−21^
*h*_max_	1.03 ± 0.05	0.95 ± 0.07	0.69 ± 0.06	319	3*10^−20^
the WTMM method					
Δ*h*	0.43 ± 0.04	0.86 ± 0.08	0.48 ± 0.05	346	5*10^−21^
*h*_min_	0.52 ± 0.05	-0.05 ± 0.02	0.11 ± 0.02	312	7*10^−20^
*h*_max_	0.95 ± 0.05	0.81 ± 0.08	0.59 ± 0.06	375	4*10^−22^

The main feature of the data obtained is that the group of patients with schizophrenia is characterized by a high degree of multifractality of EEG time series (∆*h* = 0.91 ± 0.07, MFDFA and ∆*h* = 0.86 ± 0.08, WTMM, respectively) and the presence of both anticorrelated and correlated dynamics of consecutive values of EEG ([*h*_min_; *h*_max_ ] = [0.04–0.95] by the MFDFA method and [*h*_min_; *h*_max_ ] = [-0.05-0.81] by the WTMM method).

For the group of patients with depression, the degree of multifractality is lower (∆*h* = 0.58 ± 0.06, MFDFA and ∆*h* = 0.48 ± 0.05, WTMM, respectively) than for the group of patients with schizophrenia, and there is a tendency for the singularity spectrum to shift towards anticorrelated values ([*h*_min_; *h*_max_ ] = [0.11–0.69] and [*h*_min_; *h*_max_ ] = [0.11–0.59], by MFDFA and WTMM, respectively).

For the control group, degree of multifractality (∆*h* = 0.47 ± 0.05 and ∆*h* = 0.43 ± 0.04, by MFDFA and WTMM, respectively) differs from the degree of multifractality of the EEG time series in individuals with the considered pathologies.

Thus for the site O1 over the frontal network the main differences between the multifractal properties of the healthy and pathology brain are that the EEG time series is characterized by exclusively long-term correlations for the control group, correlated and anticorrelated dynamics for the group with schizophrenia, and almost anticorrelated dynamics for the group with depression.

One-way analysis of variance revealed statistically significant differences in the averaged maximal and minimal values of the Holder exponent (*h*_min_ and *h*_max_) and the width of the singularity spectrum, Δ*h*, in the three groups examined. The statistics obtained by the Fisher *F*-criterion exceeded the critical value *F*_*crit*_ = *F*_2,28_ = 3.3 (Table 1). Significance level of the Fisher test (*p*), i.e. the maximum probability of falsely rejecting the null hypothesis of equal means, when it is true, is close to zero (Table 1).

Table 2 gives information about the averaged (over subjects) maximal and minimal values of the Holder exponent (*h*_min_ and *h*_max_) in various sites for different groups.

**TABLE 2:** Comparison of the averaged (over subjects) maximal and minimal values of the Holder exponent (*h*_min_ and *h*_max_) in various sites for different groups. The values of the Fisher test (*F*) and the significance level of the test (*p*), calculated on the basis of one-way analysis of variance are given for the multiple comparison for the width of the singularity spectra.

Sites	*control group*	*group with schizophrenia*	*group with depression*	*F*	*p*
Fz	0.35–1.15	0.21–0.91	0.31–0.71	19	7*10^−6^
F3	0.41–0.98	0.25–0.95	0.29–0.78	25	2*10^−7^
C3	0.44–1.15	0.31–1.17	0.25–0.61	21	5*10^−6^
C4	0.47–0.99	0.35–1.09	0.21–0.70	18	3*10^−6^
P3	0.53–1.08	0.03–1.12	0.07–0.55	356	6*10^−23^
P4	0.57–1.15	0.09–1.25	0.15–0.62	343	9*10^−22^
T5	0.59–1.11	0.01–1.05	0.21–0.73	327	2*10^−22^
T6	0.56–0.91	0.06–0.98	0.12–0.69	314	7*10^−22^
O2	0.51–0.95	0.04–0.95	0.13–0.75	308	2*10^−22^

The minimum values of the Holder exponent *h*_min_ are close to each other for the group with schizophrenia and the group with depression for the sites Fz, F3, C3, C4, but the maximal values of the Holder exponent *h*_max_ differ by an upward shift for the group with schizophrenia. At the same time, group differences in the [*h*_min_, *h*_max_] intervals are most typical for P3, P4, T5, T6, and O2 sites. Thus, sites over the dorsal attention and visual networks are characterized exclusively by long-term correlations of consecutive EEG values for the control group, predominantly anticorrelated dynamics for the depression group, and both correlated and anticorrelated dynamics for the schizophrenia group.

One-way analysis of variance proved statistically significant differences in the width of the singularity spectrum, Δ*h*, in all sites of the three groups examined. The values of the Fisher test (*F*) and the significance level of the test (*p*), calculated on the basis of one-way analysis of variance are given for the multiple comparison for the width of the singularity spectra. The statistics obtained by the Fisher *F*-criterion exceeded the critical value *F*_*crit*_ = *F*_2,28_ = 3.3 for the all sites. (Table 2).

Graphical results of one-way analysis of variance for comparing the average values of the width of the singularity spectrum, ∆*h*, in three groups of subjects are shown in Figure 5 (for the site C4 (Figure 5A) and the site O2 (Figure 5B). Large differences in the central lines (medians of sample values of the coefficient ∆*h*), corresponding to large values of the Fisher statistics *F*, indicate significant differences in group means.

Pairwise comparisons of the mean values of the coefficient ∆*h* obtained on the basis of multiple comparison followed by testing of paired hypotheses using the Tukey test show that for the site O2 the mean ∆*h* values for the group with schizophrenia and control group (∆*h =* 0.91 *±* 0.08 and ∆*h =* 0.44 *±* 0.04, respectively) differ at a significance level of *p* < 0.0007, for the group with depression and control group differ at the *p* < 0.0009 significance level, for the group with schizophrenia and the group with depression they differ at the *p* < 0.008 significance level.

For the site C4 these differences also exist. The smallest differences are observed for this site between the mean ∆*h* values for the group with schizophrenia and control group differ at the *p* < 0.011 significance level.

Thus, sites over the dorsal attention and visual networks are characterized exclusively by long-term correlations of consecutive EEG values for the control group, predominantly anticorrelated dynamics for the depression group, and both correlated and anticorrelated dynamics for the schizophrenia group.

Thus, the results obtained in this work, indicate, firstly, a high degree of stability of the multifractality of various EEG time series for a certain test group, and, secondly, they indicate the correlated dynamics in the analyzed sites over the dorsal attention and visual networks of the control group and predominantly anticorrelated dynamics, i.e. a significant decrease or even complete disappearance of the long–range correlations in the EEG time series for the group with depression. The EEG time series of the patients with schizophrenia are characterized by both correlated and anticorrelated dynamics of consecutive EEG values with increasing degree of multifractality in the analyzed sites over the dorsal attention and visual networks.

## 4 Discussion and Conclussions

The obtained results confirm the stability of the multifractal properties of various EEG time series, demonstrating the absence of significant differences in the spectra of the singularity внутри каждой группы for different electrode sites over the dorsal attention and visual networks.

Our results agree with the work (Popivanov et al., 2006), which shows that under different conditions for performing the visual-motor tracking task by healthy subjects (both imaginary and real visual-motor tracking), multifractal properties of the filtered EEG components are very stable for the brain activity of large brain areas, i.e. for different electrode sites. In this case, external events (task conditions) have a little effect on the results of the analysis of multifractal properties. In other words, multifractality of the healthy brain is statistically stable as well as stable its neurodynamics (Dik and Nozdrachev, 2019) and the multifractal dynamics is predominantly an endogenous property of such a self-organizing system as the human brain, which ensures its purposeful behavior (Popivanov et al., 2006).

A comparative analysis of the multifractality degree in EEG time series registered in the group of healthy subjects and the two groups of patients with mental disorders showed statistically significant differences in the singularity spectra based on the evaluation of the multifractal scaling properties of these components.

The main feature of the analyzed EEG time series of the control group is the presence of exclusively long-term correlations of consecutive values of these series. A slight shift of the singularity spectrum towards anticorrelation values is observed only in the sites over the frontal and somatomotor networks.

In contrast, the EEG time series of patients with paranoid schizophrenia have not only the long-term correlations but also the anticorrelated dynamics of consecutive values of the EEG. At the same time, a large degree of anticorrelated dynamics associated with a decrease in the minimum value of the Hölder exponent is characteristic of the sites over the dorsal attention and visual networks.

The EEG time series of patients with depression have practically the anticorrelated dynamics of consecutive values. The degree of anticorrelations increases with the transition from the frontal and somatomotor networks to the dorsal attention and the visual networks.

Thus, it can be concluded that mental disorders are correlated with impaired correlated dynamics. At the same time, the severity of brain disorders correlates with an increase in the degree of EEG multifractality.

The presence of anticorrelations of consecutive EEG values in patients with depression found in our work is consistent with the work (Bachmann et al., 2014), in which, using the method of detrended fluctuation analysis, a shift of the Hurst exponent was shown in the direction corresponding to a decrease in long-range correlations and the emergence of anti-correlation EEG dynamics in group of depressive subjects compared with the control group.

A number of studies have investigated the multifractal characteristics of filtered EEG components for a healthy brain (Popivanov et al., 2006) and fractal characteristics in the case of mental disorders (Wang et al., 2004; Raghavendra et al., 2009). The work (Wang et al., 2004) showed a decrease in the degree of correlation of successive values of the alpha and beta components of the EEG in patients with schizophrenia. However, firstly, the authors of (Wang et al., 2004) applied the method of estimating the fractality associated with the unique Hölder exponent and not with the singularity spectrum, i.e. set of the Holder exponents. Second, narrow-band data filtering can break correlations between consecutive values of time series. For this reason, such filtering was not carried out in the present work. Although the predominance of a certain rhythm in the EEG (for example, alpha or theta) may affect the fractal characteristics due to the greatest contribution of these components (Popivanov et al., 2006).

It was shown in (Raghavendra et al., 2009) that the fractal dimension of the EEG of neuroleptic-naïve, recent-onset schizophrenia subjects with positive symptoms of schizophrenia (delusions and hallucinations) was similar or higher than the fractal dimension of the EEG of the control group; this increase in fractal dimension value was absent in patients with negative symptoms of schizophrenia (apathy, lack of will). These data are consistent with the results of our work (greater degree of multifractality in patients with positive symptoms of schizophrenia compared with the control group). This agreement is also with the work (Wang et al., 2004), which also shows a significant increase of the multifractal singularity spectra in the EEG of the schizophrenic patients.

In (Alamian et al., 2022), using the wavelet leaders-based multifractal analysis (Wendt and Abry, 2007), an increase in the multifractality of the neuromagnetic (MEG) signal was shown in patients with schizophrenia in the temporal, parietal, and occipital areas compared to healthy controls. In (Racz et al., 2021), a greater degree of multifractality was found in patients with schizophrenia compared to the control group values in delta band (0.5–4 Hz) neural activity.

Due to the fact that scale-free fluctuations are considered the result of an underlying self-organized critical state of the brain that gives rise for its ability to perform large-scale reorganizations quickly in response to external/internal stimuli (Mukli et al., 2018), an increased degree multifractality in patients with schizophrenia may be associated with distorted and disorganized EEG patterns (Racz et al., 2020).

It should be noted that the results of the study of fractal dimension in patients with schizophrenia may show differences depending on the time of illness, symptoms and medication. The diversity of schizophrenia symptoms and drug treatment options, and the fact that sometimes certain combinations of drugs that help manage positive symptoms can exacerbate negative symptoms (Goff et al., 1996), make it difficult to compare results from different studies and affect the complexity of brain signals (Lee et al., 2021). In addition, differences may be due to differences in the age of patients. Indeed, it has been often reported that the properties of scale-free dynamics change with age (Churchill et al., 2016).

From a practical standpoint, multifractal dynamics often emerge from intermittent periods of larger variance due to large scale reorganizations of functional networks (Ihlen and Vereijken, 2010). An increase in the width of the spectrum and, accordingly, the degree of multifractality may be associated with increased variability in neuronal activity, which underlies excessive switching between neuronal states in patients with mental disorders (Slezin et al., 2007). This increase may reflect more random connections between neuronal activations, which may lead to cognitive impairment (Nikulin et al., 2012). The use of antipsychotics appears to reduce abnormally high EEG disorganization in patients with mental disorders (Takahashi et al., 2009).

The decrease in the degree of multifractality and finding the spectrum of multifractality in the region of anticorrelated values, which is observed in the group of patients with depression, can be interpreted as a decrease in the variability of neuronal activity associated with a decrease in the severity of mental disorders.

To sum up, further research of multifractal nature of psychiatric diseases promises to reveal new exact methods of diagnosing while it also helps to bridge the gap in the understanding of the phenomenal and neuronal nature of mental deficits**.**

## Data Availability

The raw data supporting the conclusion of this article will be made available by the authors, without undue reservation.
